# Oral Health

**DOI:** 10.1155/2012/809465

**Published:** 2012-07-05

**Authors:** Terry Fulmer, Rita A. Jablonski, Elizabeth Mertz, Mary George, Stefanie Russell

**Affiliations:** ^1^Bouvé College of Health Sciences, Northeastern University, Boston, MA, USA; ^2^School of Nursing, Pennsylvania State University, University Park, PA, USA; ^3^School of Dentistry, University of California San Francisco, San Francisco, CA, USA; ^4^University of North Carolina Chapel Hill, Chapel Hill, NC, USA; ^5^Departments of Epidemiology & Health Promotion and Periodontics, New York University, New York, NY, USA

The Institute of Medicine Report called for a greater role for nurses within the context of oral health in two recent publications, *Advancing Oral Health in America *(2011) and *Improving Access to Oral Health Care for Vulnerable and Underserved Populations* (2011). Nurses provide care for many vulnerable persons, including frail and functionally dependent older adults, persons with disabilities, and persons with intellectual and developmental disabilities. These persons are the least likely to receive necessary, health-sustaining dental care (which is distinct from mouth care). The mouth, or more accurately, plaque, serves as a reservoir for bacteria and pathogens. The link between mouth care, oral health, and systemic health is well documented; infections such as pneumonia have been linked to poor oral health. Nurses, therefore, need to reframe mouth care as oral infection control and infection control more broadly. They can provide the preventive measures that are crucial to minimizing systemic infections. Nurses in all settings can potentially provide mouth care, conduct oral health assessments, educate patients about best mouth care practices, and make dental referrals. Yet, nurses are often hesitant to do anything beyond basic oral hygiene and, even in this area, often fail to provide mouth care based on best practices. There are many reasons for this hesitancy as noted by the authors of the ten papers published in this special issue. The problems, and their solutions, can be approached from three perspectives: nursing education, practice, and research.

The root of the problem begins in basic nursing education. R. A. Jablonski asserts that nurses lack knowledge regarding basic mouth care, especially as this care pertains to older adults. In her paper, she describes the overall quality and quantity of oral hygiene content in seven major nursing fundamentals textbooks. She concludes that much of the information is incomplete, erroneous, or outdated. While R. A. Jablonski poses one plausible explanation for knowledge deficits regarding mouth care, three authors provide potential solutions. M. C. Dolce observes that nursing faculty are often ill-prepared for teaching content related to oral health, oral health assessments, and best practices in oral systemic health. She introduces the *Smiles for Life*:* A National Oral Health Curriculum* as a starting place for nursing faculty to develop their own competencies and to transmit them to their students. M. C. Dolce et al. present the *Oral Health Nursing Education and Practice *program, a national initiative whose goal is to create a nursing educational infrastructure. J. E. Hahn et al. offer an exemplar for introducing oral health content and skills pertinent to the care of elders and persons with disabilities into graduate nursing education. From the evaluation data reported in their papers, J. E. Hahn et al.'s strategy shows promise.

Education, as noted earlier, is only one problematic area when examining why nurses must take ownership of oral health and hygiene. Three of the papers in this special issue address clinical issues pertinent to oral health. K. Fisher provides an overview of best oral hygiene and dental care for persons with intellectual and developmental disabilities—a group that is at high risk for poor oral health, and ultimately, poor systemic health. M. E. McNally et al. discuss how to integrate oral care practices into organizational policy and practice in long-term care facilities in Canada. T. Fulmer and P. Cabrera argue that the primary care visit provides an optimum opportunity for the clinician to provide oral health assessment and screening. They note that while nurse practitioners are able to apply fluoride varnish to children under 19 years of age, no data exist describing this practice among nurse practitioners. 

The dissemination of research findings that inform education and practice provides the final tier towards moving nurses into accepting responsibility for the oral health and hygiene of their patients. M. R. Frazelle and C. L. Munro describe the state of the science as it pertains to toothbrush contamination. They note that existing research provides little direction in the area of toothbrush contamination or disinfection, which explains in part the lack of evidence-based nursing guidelines for toothbrush storage and decontamination. N. VanDevanter et al. offer an interesting perspective from patients regarding HIV screening during dental visits as part of nursing-dental collaboration. The patients were positive about being tested as part of the dental examination, citing knowledge of HIV status and convenience of testing as two major benefits. Finally, S. Williamson et al. compared 27 cytokines in plasma samples, passive drool samples, and filter paper samples from 50 subjects. They found that relationships were dependent upon the specific biomarker. This research can help inform future studies and clinical practices related to cytokine measurement, especially those cytokines implicated in illnesses with oral system associations.

We hope that this special issue provides the catalyst to help nurses take ownership of their role in health promotion as it pertains to oral health. Mouth care and oral hygiene are more than an activity of daily living; they are imperative to safe and quality care. 

*Terry Fulmer* *Terry Fulmer* *Rita A. Jablonski* *Rita A. Jablonski* *Elizabeth Mertz* *Elizabeth Mertz* *Mary George* *Mary George* *Stefanie Russell* *Stefanie Russell*

